# High-frequency transcription leads to rapid R-loop formation

**DOI:** 10.1016/j.jbc.2025.108514

**Published:** 2025-04-16

**Authors:** Bradleigh Palmer, Chun-Ying Lee, Leya Yang, Tapas Paul, Sua Myong

**Affiliations:** 1 Department of Biophysics, Johns Hopkins University, Baltimore, Maryland, USA; 2 Program in Cellular and Molecular Medicine, Boston Children's Hospital, Boston, Massachusetts, USA

**Keywords:** EMSA, R-loop, single-molecule FRET, stochastic simulation, transcription frequency, transcription rate

## Abstract

R-loops are transcriptionally generated three-stranded nucleic acid structures where the mRNA hybridizes with template DNA, leaving a displaced single-stranded non–template DNA loop. Previously, we demonstrated that R-loop and subsequent G-quadruplex formation upregulate transcription. However, the mechanistic basis of how transcription activity generates R-loop formation is unknown. Here, we investigate the kinetics of transcription and its impact on R-loop formation using single-molecule FRET and EMSA. We show that R-loop formation is tuned by the frequency and the rate of transcription, controlled by the RNA polymerase and NTP concentrations, respectively. We provide a plausible mechanism in which gradually increasing the duration of the promoter opening leads to the R-loop formation. Through stochastic simulation, we demonstrate that the frequency of transcription primarily governs R-loop formation. This work highlights the intricate balance between transcription dynamics and R-loop formation, providing new insights into the structure–function relationship.

R-loops are intriguing three-stranded nucleic acid structures consisting of RNA:DNA hybrid, forming when nascent RNA hybridizes with the template DNA and displaces the non–template DNA strand (1, 2). R-loop formation can occur at telomeres and centromeres, during double-strand break repair, and during transcription through *cis*- or *trans*-mechanisms (3, 4). R-loops are often associated with GC-rich regions, where guanine clustering in the non–template strand facilitates R-loop initiation (5, 6, 7, 8). Many techniques have been reported to detect R-loops in the genome, such as reverse chromatin immunoprecipitation and DNA–RNA immunoprecipitation sequencing, which utilize inactive RNase H or an R-loop antibody, respectively (9, 10, 11). R-loops are pivotal in various cellular functions, including class-switch recombination in B cells, transcriptional regulation (both silencing and activation), and transcription termination (3, 12, 13, 14, 15, 16). However, unscheduled R-loop formation can induce aberrant gene regulation and genome instability, potentially leading to DNA damage, neurodegeneration, and cancer (17, 18, 19, 20). Thus, the formation of R-loops is tightly regulated within cells.

Transcription-related R-loops can be categorized into two types with distinct characteristics: promoter-proximal (type I) and elongation-associated R-loop (type II) (21), and a third type may exist and be related to termination mediated by senataxin (22, 23, 24). Many studies of R-loop profiling have demonstrated that type I R-loops are enriched at active promoter regions and correlated with high transcription levels (9, 25, 26, 27), but they may also cause RNA polymerase (RNAP) pausing (28). However, scientists have struggled to figure out how R-loop forms during transcription. The precise kinetics of R-loop formation and the relationship between transcription activity and R-loop forming propensity remain elusive.

We employed T7 RNAP, a single-subunit bacteriophage RNAP known for its well-characterized structure and kinetics. It is an ideal candidate for *in vitro* transcription studies (29, 30, 31, 32). R-loop formation has been reported in G-rich templates by *in vitro* T7 RNAP transcription (33, 34). Our previous work has shown that R-loop formation precedes the formation of G-quadruplexes (G4s), noncanonical secondary structures in guanine-rich DNA and RNA (35, 36), and G4 formation in the non–template strand of DNA further stabilizes R-loop structure (37), providing a suitable platform to investigate the kinetics of R-loop formation during transcription. The current model suggests that R-loop formation is a probabilistic event that occurs after a single transcription cycle (38). A single-molecule study reported that the probability of R-loop formation by a single turnover transcription is about 0.26% in the presence of a potential G4-forming sequence (PQS) at a non–template DNA strand (39). However, our previous results and their data showed that the fraction of R-loops increased significantly more when multiple-turnover reactions were allowed (34, 37, 39), suggesting that the kinetics of R-loop formation depends on the RNAP loading frequency and RNA synthesis rate.

We sought to investigate the kinetics of R-loop formation with respect to the transcription activity. We used single-molecule and biochemical methods to monitor R-loop formation during transcription. Our results reveal that R-loop formation is influenced by the frequency and speed of transcription, which are controlled by RNAP and NTP concentrations, respectively. We propose that the high transcription frequency, which prolongs progressively the opening of the promoter site, facilitates R-loop formation. Through single-molecule analysis and stochastic simulation, we found that the effective intrinsic probability of R-loop formation correlates with high transcription frequency.

## Results

### R-loop formation quantified by single-molecule FRET and EMSA

**Figure 1:**
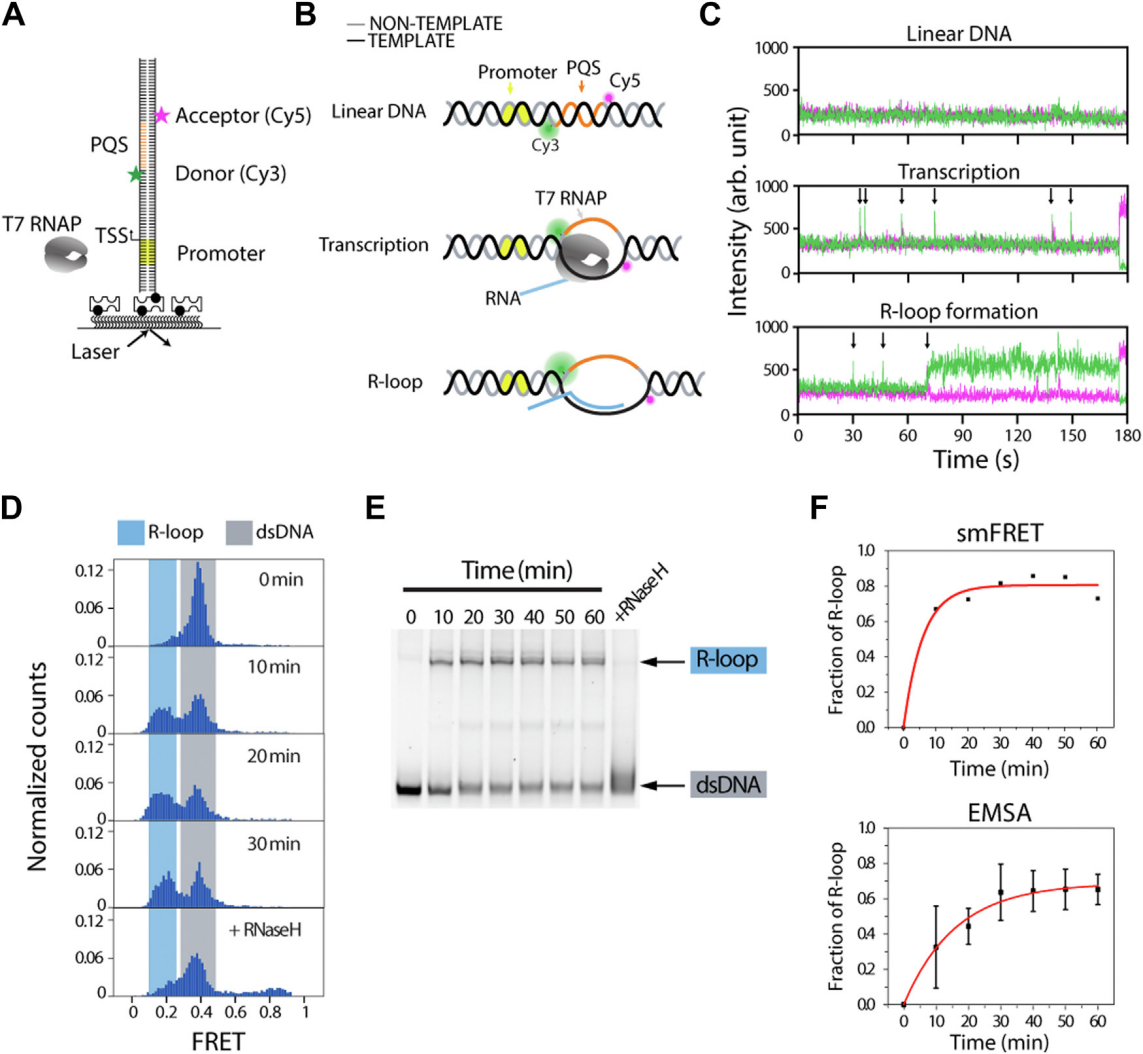
**R-loop formation was visualized and quantified by smFRET.***A*, DNA construct for single-molecule experiments. *B*, schematics of transcription states and FRET transition. FRET construct produces a 0.4 FRET value when DNA is linear; upon R-loop formation, the dyes are further apart producing a 0.2 FRET. *C*, representative single-molecule traces of DNA in linear state, during transcription, and a molecule that shifts to R-loop state. *D*, FRET histogram with 10 min incubation intervals using 1 mM NTP and 1 μM RNAP. *E*, EMSA gel with 10 min incubation intervals using 1 mM NTP and 1 μM RNAP. *F*, quantification of smFRET and EMSA showing R-loop fraction *versus* time. *D* and *F*, smFRET, a single representative histogram was quantified, n = 3 independent measurements were taken. 1 mM NTP and 1 μM RNAP are used. *E* and *F*, the EMSA gel is a representative image, and the quantification is from n = 3 independent gels. *F*, both curves were fit with an exponential function. RNAP, RNA polymerase; smFRET, single-molecule FRET.

To investigate the kinetics of R-loop formation mediated by transcription activity, we utilized T7 RNAP for *in vitro* transcription in single-molecule and ensemble experiments based on our previous study (37). We prepared FRET-labeled and biotinylated DNA substrates to be tethered to single-molecule surface (40) (Fig. 1*A*). The DNA construct contains a T7 promoter with FRET-paired dyes, Cy3 and Cy5, surrounding a PQS (Fig. 1*A*), which enables observation of R-loops during transcription elongation (Fig. 1*B*). The constant intensity, at the beginning, indicates that the DNA helical structure remains unchanged (Fig. 1*C*, *top*). The spikes of fluorescence that appear upon adding RNAP and NTP (Fig. 1*C*, *middle*) indicate successive RNAP molecules translocating through the FRET detection region. Briefly, the increase in Cy3 fluorescence is due to PIFE (protein-induced fluorescence enhancement), reporting on the incoming RNAP near the Cy3 dye (41). We chose cMyc PQS because of its high propensity to form G4 and R-loop structures (Fig. S1) during transcription (37). The R-loop formation can be detected as a sudden FRET decrease because of the RNA:DNA hybrid, which increases the distance between the two dyes (Fig. 1*C*, *bottom*). Therefore, the R-loop formation can be quantified by the FRET histogram, where linear DNA has a FRET peak at 0.4, whereas the R-looped DNA peak shifts to a FRET value of 0.2 (Fig. 1*D*). This shift in FRET value occurs because of the stretching of the non–template DNA strand during R-loop formation. The R-loop FRET peak was confirmed by performing RNase H digestion, which digests the RNA within the R-loop, thus removing the FRET peak of 0.2 (Fig. 1*D*, *bottom*). Our results suggest that the R-loop state progressively accumulates during transcription and is only disturbed by removing the hybrid RNA strand.

Next, we quantified R-loop formation using an EMSA. The same DNA construct designed for our single-molecule FRET (smFRET) experiments was used for the EMSA analysis. Samples were collected in 10-min intervals from a 1-hour-long measurement and treated with 0.1% SDS to denature and remove the RNAP from DNA. The dsDNA band shifts upward as R-loops retard the electrophoretic mobility because of the increased molecular weight and hydrodynamic radius (Fig. 1*E*). As in the smFRET experiment, the R-loop band progressively increases over the course of transcription and disappears upon treatment with RNase H, confirming that the upshifted bands represent R-looped structures. The R-loop formation for smFRET and EMSA was quantified and normalized (Fig. 1*F*). Both assays showed a similar pattern, although a lower rate of R-loop formation was observed in EMSA because of a higher DNA concentration (10 nM) compared with smFRET (0.2 PM), resulting in a lower RNAP:DNA ratio. However, we found that both assays led to a similar plateau of R-loop formation (∼70%). This phenomenon suggests that a kinetic factor is likely involved in R-loop formation.

Transcription is a multistep reaction consisting of RNAP binding to the promoter, preinitiation with structural changes, initiation of RNA synthesis, elongation, and termination. It is challenging to determine which step or steps are responsible for the R-loop formation. Importantly, we previously demonstrated that R-loops form after multiple rounds of transcription events, which raises an intriguing question about how the kinetics of transcription gives rise to the R-loop formation (Equations 1 and 2 below). The first equation defines the RNAP binding, whereas the second assumes NTP incorporation as the rate-determining step. The inverted reaction arrows indicate the transcription events where DNA remains duplexed without leading to the R-loop formation. To simplify the model, we define the R-loop in the equations as a stable structure, thus no reverse reaction is included.

(1) DNA+RNAP⇌Transcription→Rloop

(2) DNA:RNAP+NTP⇌Transcription→Rloop

### R-loop formation is dependent on RNAP and NTP concentration

**Figure 2:**
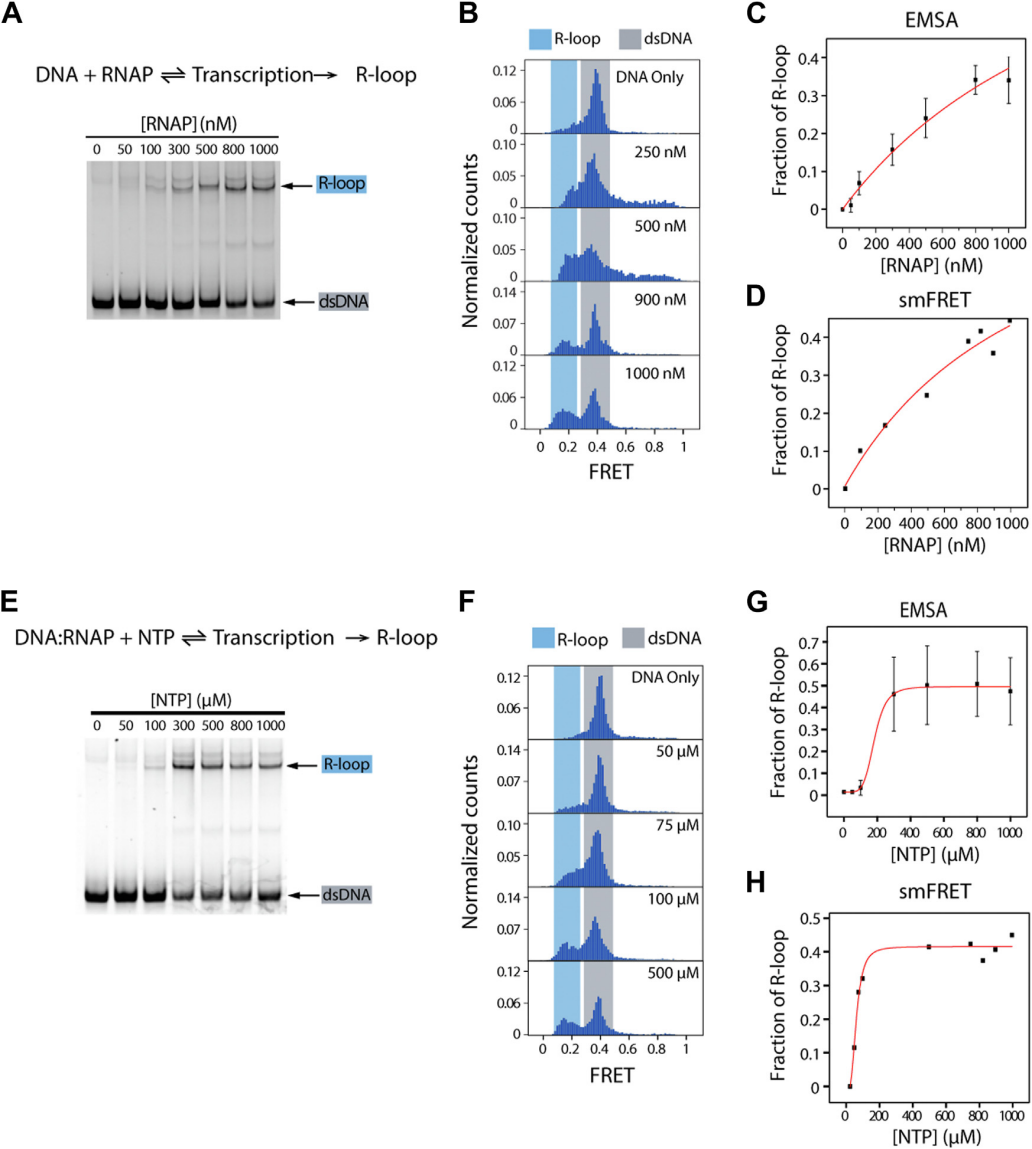
**R-loop formation increases as RNAP concentration or NTP concentration increases.***A*, EMSA gel of RNAP titration after 10 min of transcription with 1 mM NTP. *B*, FRET histogram of select RNAP concentrations after 10 min of transcription with 1 mM NTP. *C*, quantification of EMSA. *D*, quantification of FRET histogram. *A* and *C*, EMSA gel is a representative image, and quantification is from n = 3 independent gels. *B* and *D*, a single representative histogram was quantified, n = 2 separate measurements were taken. *C* and *D*, curves were fit with Equation 3, and the *K*_*RNAP/R-loo*p_ were 1.68 ± 0.09 μM and 1.2 ± 0.6 μM for EMSA (*C*) and smFRET (*D*), respectively. *E*, EMSA gel of NTP titration after 10 min of transcription with 1 μM RNAP. *F*, FRET histogram of select RNAP concentrations after 10 min of transcription with 1 μM RNAP. *G*, quantification of EMSA. *H*, quantification of FRET histogram. *E* and *G*, the EMSA gel is a representative image, and quantification is from n = 3 independent gels. *F*, a single representative histogram was quantified, n = 2 separate measurements were taken. *G* and *H*, curves were fit with a hill function, and the *K*_*NTP/R-loop*_ were 183 ± 22 μM (n = 5 ± 1) and 61 ± 6 μM (n = 3.2 ± 0.8) for EMSA (*G*) and smFRET (*H*), respectively. RNAP, RNA polymerase; smFRET, small-molecule FRET.

First, we asked whether the ratio of RNAP to DNA determines R-loop formation (Equation 1). We titrated RNAP concentration at a fixed NTP concentration of 1 mM. The reactions were terminated to collect the samples every 10 min for a total of 60 min. The reaction progression was measured by EMSA (Fig. 2*A*) and smFRET (Fig. 2*B*). The R-loop fractions were quantified and fitted to determine the concentration *K*_*RNAP/R-loop*_ by Equation 3 below, which represents the RNAP concentration at which the half-maximal level of R-loop formation occurs within 10 min. The values were determined as 1.68 ± 0.09 μM and 1.2 ± 0.6 μM for EMSA and smFRET, respectively (Fig. 2, *C* and *D*). The concentrations were one to two orders of magnitude higher than the *K*_*d*_ reported for T7 RNAP binding to DNA (42), indicating that RNAP binding alone cannot induce R-loop formation and further transcription activity is required. The result shows that the R-loop fraction increases as a function of RNAP concentration, demonstrating that R-loop formation is promoted at higher RNAP concentrations. Since the NTP concentration was unchanged in all conditions, the frequency of RNAP loading to DNA, that is, higher frequency of transcription, likely leads to higher R-loop formation.

(3) $$Fraction\;of\;R-loop,\theta =\frac{\left[RNAP\right]}{K_{RNAP/R-loop}+\left[RNAP\right]}$$

Next, we performed transcription reactions in varying NTP concentrations to test how incorporating NTP controls the transcription rate and affects R-loop formation (Equation 2). Here, we do not distinguish multiple states of NTP hydrolysis to simplify the kinetic description and focus on the rate of RNA synthesis. Similar to the RNAP titration, we titrated the NTP concentration at a fixed T7 RNAP concentration of 1 μM. EMSA and smFRET revealed that increasing NTP concentration resulted in higher R-loop formation (Fig. 2, *E* and *F*). Since the process entails a multiple-turnover reaction, the fractions of R-loops were quantified and fitted using the Hill function (Equation 4), where *K*_*NTP/R-loop*_ represents the NTP concentration required to induce 50% R-loop formation within 10 min. The resulting values were 183 ± 22 μM (n = 5.3 ± 1.2) and 61 ± 6 μM (n = 3.2 ± 0.8) for EMSA and smFRET, respectively (Fig. 2, *G* and *H*). Again, the difference between EMSA and smFRET is likely due to the lower RNAP:DNA ratio in EMSA compared with smFRET. Our results indicate that the fraction of the R-loops can also be a function of NTP concentration, and the Hill coefficient suggests that R-loop formation requires multiple rounds of transcription.

(4) $$Fraction\;of\;R-loop,\theta =\frac{{\left[NTP\right]}^{n}}{{K_{NTP/R-loop}}^{n}+{\left[NTP\right]}^{n}}$$

So far, the ensemble analysis of EMSA and smFRET revealed that both RNAP and NTP concentrations affect the kinetics of R-loop formation. While RNAP concentration determines the binding rate of RNAP on DNA, NTP concentration controls the elongation rate, both factors contribute to the overall transcription activity.

### R-loop–forming DNA exhibits a higher frequency and higher rate of transcription

**Figure 3:**
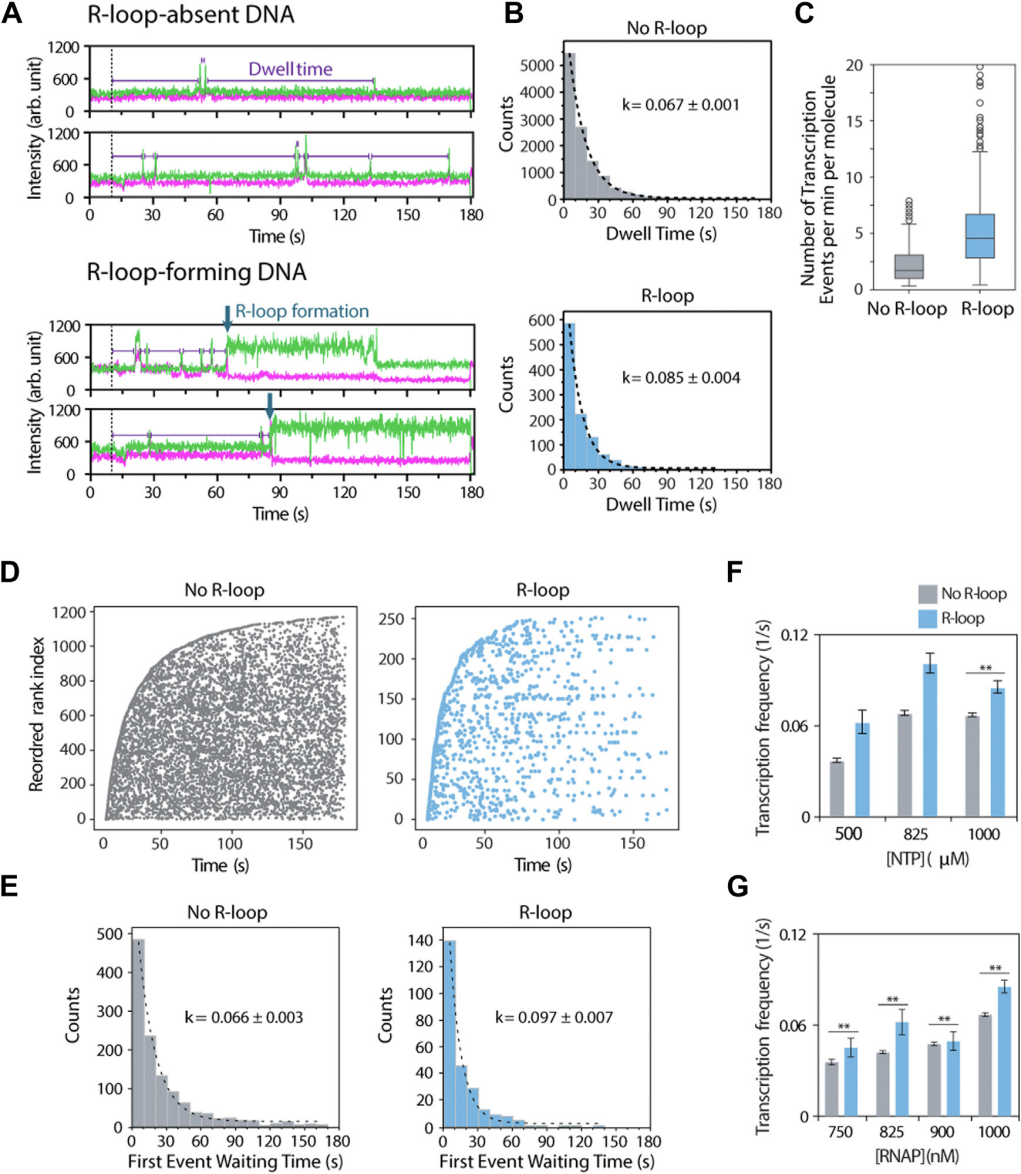
**R-loop–forming molecules have higher RNAP turnover.***A*, representative traces of molecules that do not and do form R-loops. Dwell times are indicated by *purple lines*, and R-loop formation is indicated by *blue arrows*. *B*, time between transcription events fits with an exponential decay function, *p* value = 0.0022. *C*, number of transcription events per minute per molecule, normalized by using the time to R-loop for R-loop–forming traces or the full trace length for non–R-loop forming traces, *p* value = 2.2 × 10^−16^, Mann–Whitney *U* test. *D*, times of all transcription events plotted for each molecule observed. The molecules were aligned by the order of first-event time, defined in the *y*-axis of reordered rank. *E*, time to first transcription event fit with an exponential decay function, *p* value = 0.0085. *F*, RNAP transcription frequency of various NTP titrations. *G*, RNAP turnover rate of various RNAP titrations. *A*–*E*, 1 μM RNAP and 1 mM NTP are used. *B*, *E*–*G*, RNAP transcription frequency is calculated by fitting dwell times with an exponential decay function. Times were binned, and rank of the bins was compared using a Mann–Whitney *U* test. *B*–*G*, molecule counts for each condition can be found in Appendix 3. *F* and *G*, 1 μM RNAP is used for NTP titration, and 1 mM NTP is used for RNAP titration. Data are presented as value ± SD. ∗*p* ≤ 0.05, ∗∗*p* ≤ 0.01 (Mann–Whitney *U* test). RNAP, RNA polymerase.

Next, we wanted to ask if there is an intramolecular factor, that is, the DNA molecule itself, that impacts the formation of the R-loop. One of the advantages of the smFRET platform is its ability to detect individual DNA molecules undergoing multiple rounds of RNAP binding, subsequent transcription, and R-loop formation. Multiple spikes of the fluorescence signal in the smFRET trace report on the successive transcription events (Fig. 1*C*), from which we can collect time intervals to determine the transcription frequency. In addition, we categorized the DNA molecules into two groups: R-loop-absent DNA and R-loop-forming DNA within the first 3 min of transcription activity (Fig. 3*A*). The transcription frequency was computed by fitting the convolution curve of all the interval times to the first exponential decay (Fig. 3*B*). For the R-loop–forming DNA, the number of transcription events was divided by the time it took for R-loop formation. For R-loop–absent DNA, the number of transcription events was divided by the total observation time of 180 s (Fig. 3*C*). Interestingly, we found that R-loop–forming DNA had a higher transcription frequency than R-loop–absent DNA. We further aligned all the DNA traces based on the time of the first transcription event, from fastest to slowest, to visualize the exponential dependence of the transcription reaction (Fig. 3*D*). Intriguingly, our analysis revealed that the first transcription event occurred more quickly in R-loop–forming DNA than in R-loop–absent DNA (Fig. 3, *D* and *E*). Strikingly, this difference was observed in all the titration conditions (Fig. 3, *F* and *G*). Two potential scenarios may explain our results. First, a subpopulation of DNA may exhibit an energy state favorable for R-loop formation. This mechanism posits that the energy state determines whether a DNA molecule will form an R-loop. Second, the formation of the R-loop is an independent event with a specific intrinsic probability. In this case, a DNA molecule undergoes conformational fluctuations with a certain probability of being in a conducive or nonconducive conformation for R-loop formation. Both models suggest that DNA conformation is inhomogeneous at any given time: different DNA molecules may have stably heterogeneous states (first model), or they undergo dynamic fluctuations with a specific equilibrium constant (second model).

**Figure 4:**
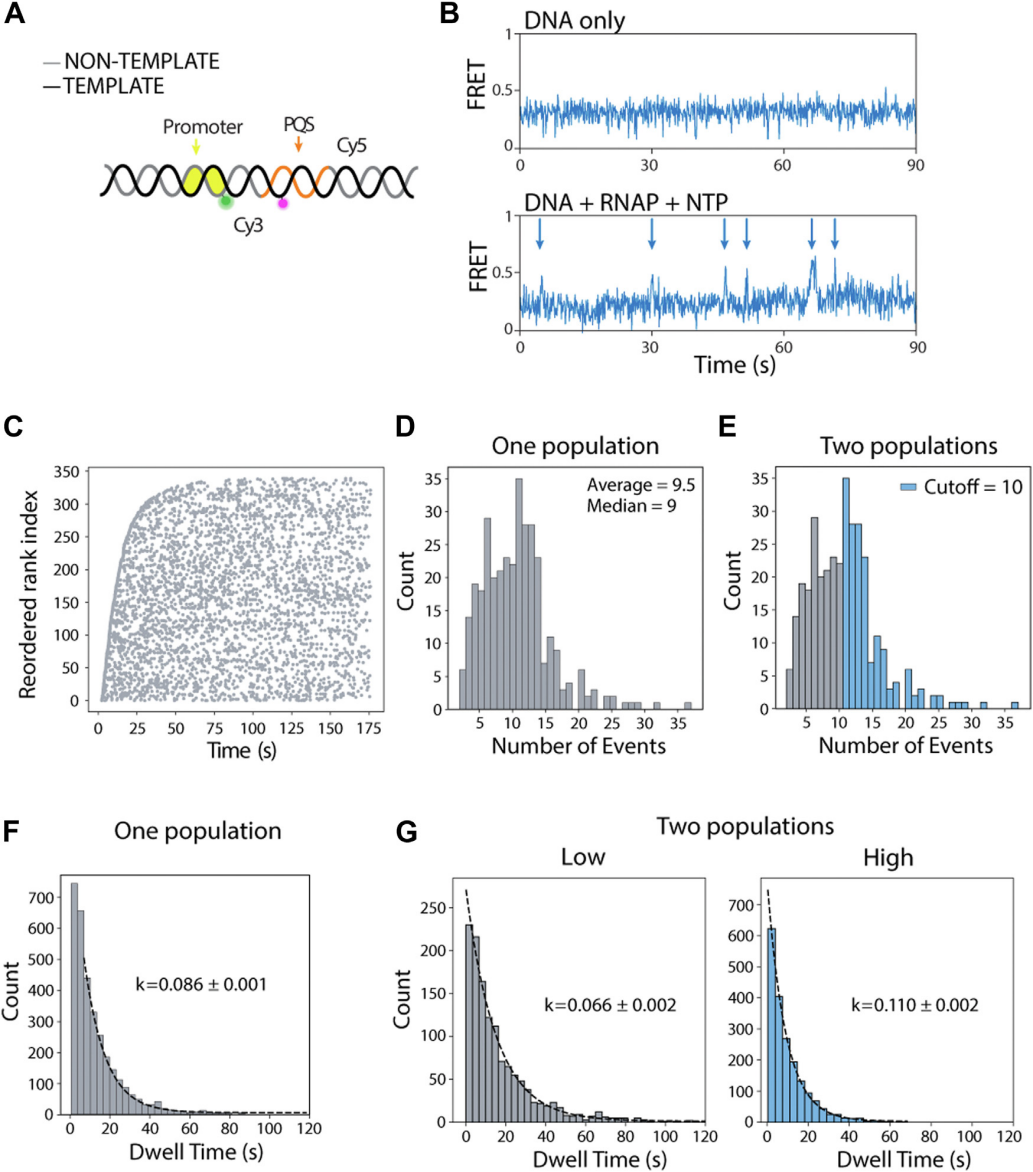
**FRET construct for monitoring transcription initiation.***A*, FRET construct with donor at promoter and acceptor at downstream. This pair location can detect the structural change of initiation. *B*, example of FRET traces to demonstrate the FRET changes in the presence of RNAP and NTP. The *arrows* indicate individual transcription initiations. *C*, time of all initiation events plotted for each molecule observed. The molecules were aligned by the order of first-event time, defined in the *y*-axis of reordered rank. *D* and *E*, the distribution of numbers of events per molecule. *D*, one population case and (*E*) two population cases. The cutoff is used to divide the molecules into a low and high number of events. *F*, time between initiation events, fitted with first-order exponential decay for all the events. *G*, time between initiation events, separated into “low” and “high” defined in (*E*). The curve was fitted with first-order exponential decay. RNAP, RNA polymerase.

To test whether the DNA population is heterogeneous, we used a different FRET-paired construct with dyes positioned across the promoter (Fig. 4*A*), which is suited for detecting transcription initiation (Fig. 4, *B* and *C*) but not the R-loop formation. If two distinct DNA populations exist, we would expect to observe two different initiation frequencies. The distribution of initiation events showed an average of 9.5 events within 3-min observation window (Fig. 4*D*). Based on a cutoff of 10 events, we found that DNA molecules could be potentially categorized into two groups (Fig. 4*D*). The overall initiation frequency was 0.086 ± 0.001 s^−1^ (Fig. 4*F*). However, applying the cutoff allowed us to separate the data into two groups with different initiation frequencies. The high-frequency group exhibited a rate approximately twice as high as the low-frequency group (Fig. 4*G*). Although this construct does not directly report on R-loop formation, the observation of two distinct initiation frequency groups suggests that different subpopulations of DNA may have varying levels of transcription initiation, which is likely linked to the propensity of R-loop formation.

**Figure 5:**
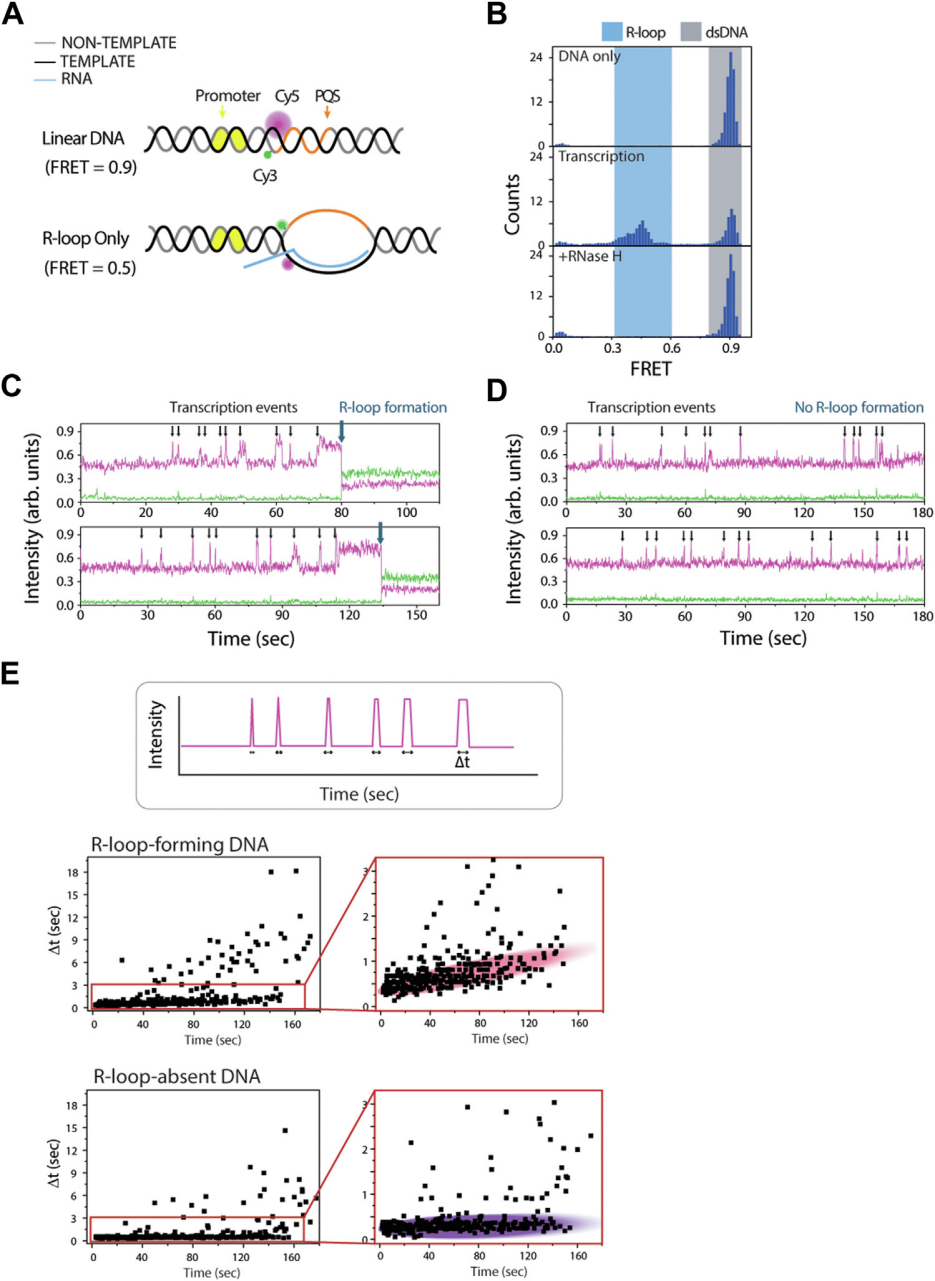
**FRET construct for detecting post-transcriptional DNA annealing.***A*, FRET construct with both dyes located upstream of PQS but at separated strand. The position of dyes was sensitive to the transcription bubble. *B*, FRET histogram shows the shift of stable R-loop formation. *C* and *D*, representative time traces of transcription bubble opening and closing. The transcription events were indicated by *black arrows*, and the transition of R-loop was indicated by *blue arrow*. *C*, R-loop formation DNA. *D*, R-loop-absent DNA. *E*, distribution of bubble opening time interval along with time trajectory. *Top*, the quantification of time interval was defined as depicted. *Mid*, R-loop formation DNA. The expanded figure shows the increase of opening increases at later time points, suggesting the DNA structure became unstable after multiple transcription turnover. *Bottom*, R-loop–absent DNA. There is no clear significant increase in opening time. PQS, potential G4-forming sequence.

Next, we sought to determine whether successive transcription events induce progressive changes in the transcription bubble formation and whether such alterations are correlated with the R-loop formation. To probe the transcription bubbles directly, we used another FRET-paired construct with the dyes conjugated immediately juxtaposed across the two strands (Fig. 5*A*), such that the DNA exhibits high FRET. A mid-FRET population appeared while the transcription continued and was removed by RNase H treatment, indicating that the mid-FRET represents the R-loop formation (Fig. 5*B*). Multiple rounds of transcription were visualized as continuous red spikes, and the high FRET was converted to mid-FRET after R-loop formation (Fig. 5*C*). The red signal increase is due to a PIFE effect of a Cy3 dye, which is transferred to Cy5 when in high FRET configuration (unpublished observation). Multiple rounds of transcription (red spikes) were also observed in both R-loop–forming and R-loop–absent molecules. The R-loop–absent molecules do not exhibit a transition to mid-FRET within 180 s (Fig. 5*D*). Therefore, the duration of the red signal spike indicates the duration of the transcription bubble open state before the DNA returns to the duplex state (Fig. 5*E*, *top*). Our result shows that for the R-loop–forming molecule, the duration of red spikes progressively increases as a function of transcription time. This indicates that successive transcription events may progressively destabilize the DNA structure at the promoter site, facilitating R-loop formation (Fig. 5*E*, *middle*). On the other hand, the reannealing time for the R-loop–absent molecule does not increase significantly, indicating the DNA structure remains relatively stable (Fig. 5*E*, *bottom*).

Our single-molecule analysis from all three constructs demonstrates that the same DNA construct may exhibit intrinsically diverse conformational states during transcription reactions. This suggests the potential for transitions between different DNA subpopulations, even without transcription, influenced by different structural and energy states. For example, local structural differences in the promoter region may interfere with RNAP binding and the subsequent opening of the transcription bubble. Once transcription is initiated, intermolecular interactions between RNAP, DNA, and NTP incorporation, driven by concentration levels, control the transcription kinetics. Specifically, as more transcription occurs, the intramolecular DNA conformation becomes less stable. This is likely due to incomplete reannealing after the first round of transcription (*i.e.*, DNA is preopened for the second RNAP to bind). This instability likely leads to a sufficiently loose DNA state for R-loop formation. Consequently, both models are likely correct and collaboratively impact the kinetics of R-loop formation.

### R-loop formation regulated by probability and active DNA state

**Figure 6:**
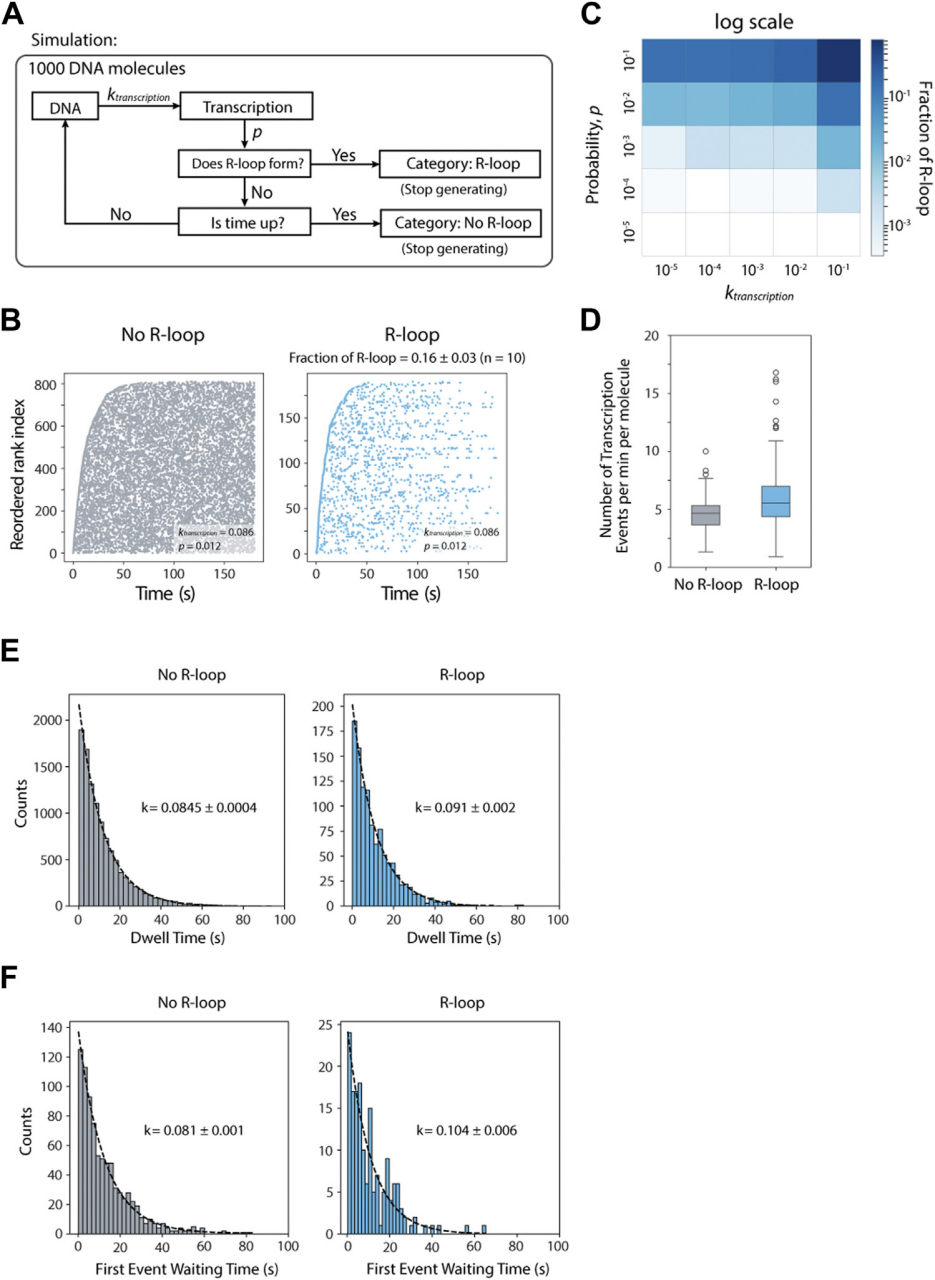
**Simulation model of R-loop formation at single-molecule level.***A*, model schematic showing independent transcription events. Transcription frequency was determined by *k*_transcription_, obtained from experimental data. R-loop formation depends on the probability (*p*). *B*, representative of transcription event plots, aligned with order of first-event time and defined in *y*-axis of reordered rank. The transcription was simulated by *k*_transcription_ = 0.086 and probability (*p* = 0.012), in which the fraction of R-loop was 16% ± 3% (10 times simulation) for a 180 s time window. *C*, R-loop fraction simulated by varying *k*_transcription_ and probability. Both *k*_transcription_ and *p* were varied from 10^−5^ to 10^−1^. The heat map indicates probability impacted on R-loop formation more than *k*_transcription_. *D*, representative of number of transcription events by simulation. *E*, representative of dwell time distribution by simulation. The fitting rate was averaged from 10 independent simulations. *F*, representative of the distribution of first event waiting time. The fitting rate was averaged from 10 independent simulations.

To further understand the underlying mechanism of R-loop formation, we simulated the cotranscriptional R-loop forming kinetics using a stochastic transcription model (Fig. 6*A*). In this model, we set two parameters, _transcription_ (*k*_tx_) and probability (*p*), where *k*_tx_ was used to determine the frequency of individual transcription events, and *p* indicated the intrinsic probability of R-loop forming for each transcription event. To simulate our single-molecule experiment, we fixed the *k*_*tx*_ as 0.086 s^−1^, the transcription frequency obtained from the single-molecule experiment (Fig. 4). Moreover, given a simulation time (180 s), each transcription event has a constant probability (*p*) of forming an R-loop structure. Once an R-loop forms, the simulation is stopped irreversibly. Each simulation consisted of 1000 independent molecules, comparable to the number of molecules observed in our single-molecule experiments (Fig. 3).

First, we demonstrate that our simulation successfully generates stochastic transcription events. Specifically, the single exponential dependence of the first transcription time (Fig. 6*B*) closely resembles our experimental data (Fig. 3*D*). Moreover, our experimental data showed that 17.8% of the molecules form an R-loop within a 180-s observation time, a result that was recapitulated by the simulation with *p* = 0.012. Next, we examined the contribution of *k*_*tx*_ and *p* to the fraction of R-loop formation. Since the single-molecule experiments could only detect a narrow range of *k*_*tx*_ from 0.05 to 0.1 s^−1^ by titrating RNAP and NTP concentration (Fig. 3, *F* and *G*), we varied both *k*_*tx*_ and *p* over a wider range, from 10^−5^ to 10^−1^. Indeed, the fraction of R-loop showed strong dependence on *p*, with only minor sensitivity to *k*_*tx*_ (Fig. 6*C*). Furthermore, we analyzed the simulation properties with *k*_*tx*_ = 0.086 s^−1^ and *p* = 0.012 to calculate the number of transcription events per minute (Fig. 6*D*), the transcription frequency for all the events (Fig. 6*E*), and the rate of first transcription event (Fig. 6*F*) based on the "R-loop–forming" and "R-loop–absent" groups. Surprisingly, with constant *k*_*tx*_ and *p*, the R-loop–forming group always exhibited higher frequency and higher transcription rate throughout multiple simulation rounds, which was entirely consistent with our experimental observations (Fig. 3, *B*, *C* and *E*).

**Figure 7:**
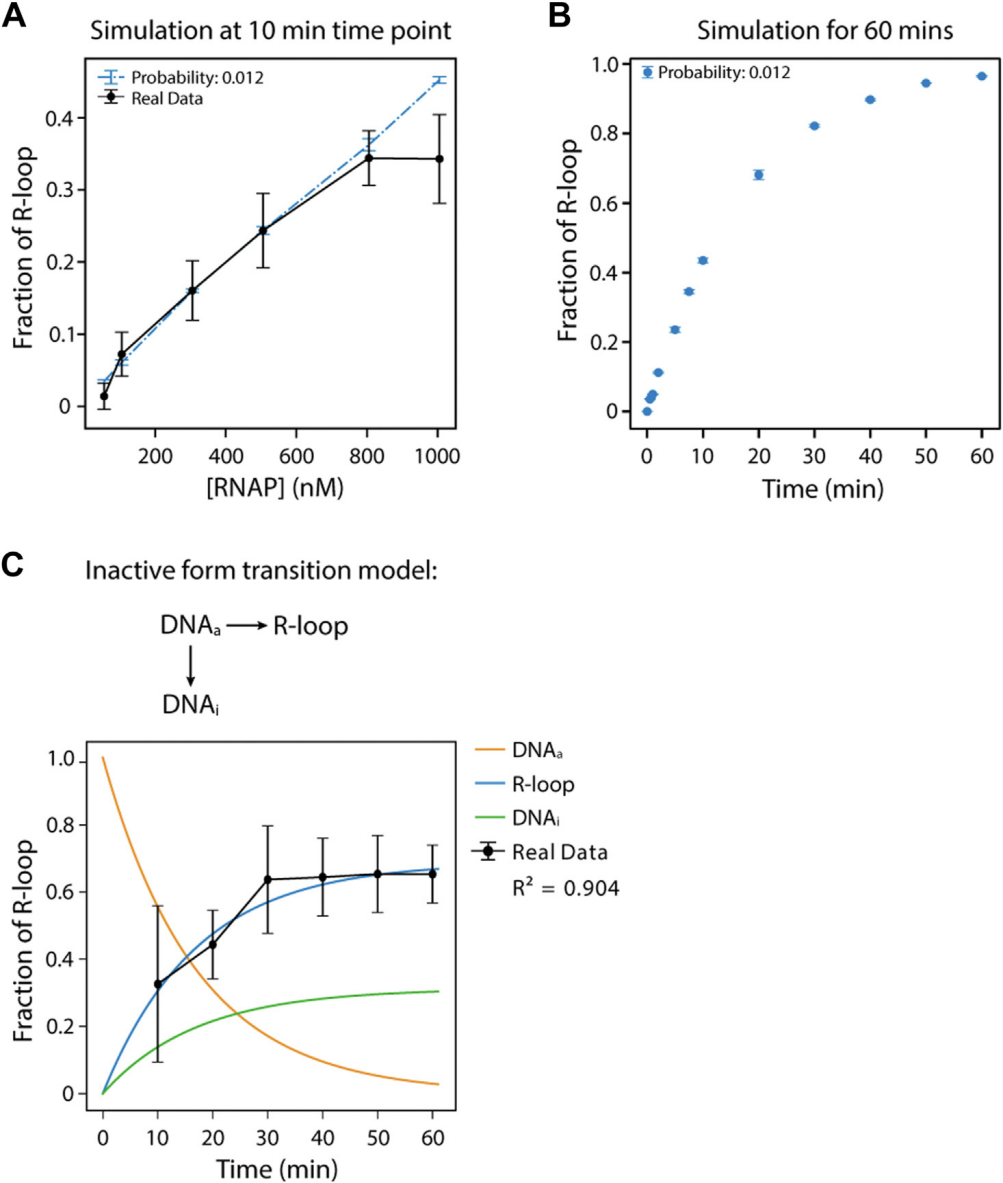
**DNA state transitions to an inactive state competes with R-loop formation.***A*, simulation of RNAP titration with probability (*p* = 0.012). The real data were calculated from EMSA. The simulation results were averaged from 10 independent simulations. *B*, simulation of R-loop formation for time point, showing 97% R-loop forms at 60 min. The simulation results were averaged from three independent simulations. *C*, ensemble simulation of competition model. The increasing population of DNA_i_ demonstrated that R-loop reaches fraction plateau around 70% instead of nearly 100%. RNAP, RNA polymerase.

With intrinsic probability *p* recognized to be the pivoting factor in R-loop formation, we found *p* = 0.012 well recapitulates the RNAP titration data from EMSA (Fig. 2*A*) when extending the observation time to 10 min (Fig. 7*A*). We observed that this specific *p* value also led to the same R-loop formation rate as seen in the smFRET experiments. However, the stochastic transcription model (Fig. 6*A*) predicts that, given enough time, all the constantly transcribed DNA will eventually form an R-loop: for instance, the simulation showed that the fraction of R-loop reached 97% by simulating for 60 min (Fig. 7*B*). In comparison, our experiments showed approximately 70% R-loop formation (Fig. 1*F*), suggesting that another kinetic event modulates the transition. Hence, we first examined whether the NTP consumption causes the plateau in R-loop fraction by estimating the NTP concentration and reaction rate over time (Fig. S2). As expected, the 1 mM NTP concentration is sufficient to support a high transcription rate of up to 60 min for the low DNA concentration (10 nM for EMSA and subnanomolar for smFRET), indicating that NTP depletion is unlikely to be the cause of the discrepancy.

Since DNA may dynamically transition to a different conformation, we hypothesized a status exchange within the DNA population, where the DNA transitions between R-loop active (DNA_a_) and R-loop inactive (DNA_i_) state. Here, we propose a competition ensemble model (Fig. 7*C*) in which all the DNAs initially start from an R-loop–proficient state (DNA_a_) in the course of transcription. Over time, a subpopulation of the DNA transitions to DNA_i_ state, which is transcription proficient yet R-loop deficient. We also noted that the transition from the active to inactive state is primarily irreversible as the backward reaction rate is negligible compared to the forward rate (data not shown). The revised simulation results based on the competition model fully recapitulated the experimental data, suggesting that the transition from the active to the inactive state is likely responsible for the finite R-loop plateau, even though we could not directly distinguish between the two DNA states.

To sum up, the kinetics of R-loop formation during transcription can be inspected from three perspectives: 1) ensemble intermolecular interactions that are affected by transcription frequency and speed *via* modulating the RNAP and NTP concentration; 2) intrinsic probability of R-loop formation at each transcription event, which could be impacted by many factors, including R-loop prone motif, DNA supercoiling, temperature, and the length of DNA substrate; 3) intramolecular properties, for example, locally unstable structure after transcription, and energy state that favors RNAP binding and transcription.

## Discussion

R-loop has been reported to be highly correlated with active transcription sites and PQSs. However, the mechanism of R-loop formation remains unclear. Previous research has proposed that the R-loop is a byproduct of transcription with an extremely low probability (38). Here, we utilized single-molecule experiments and ensemble methods to investigate the kinetics underlying R-loop formation during transcription (Fig. 1). Our results indicate that R-loop formation increases over time and is impacted by the RNAP concentration, which controls RNAP occupancy on DNA, and the NTP concentration that modulates the speed of transcription (Fig. 2). We showed that transcription activity is significantly faster in R-loop–forming DNA compared with R-loop–absent DNA (Fig. 3), and the successive transcription destabilizes the DNA structure (Fig. 5). Furthermore, we simulated a transcription model and demonstrated that R-loop formation is primarily controlled by intrinsic probability and regulated by the transcription rate and frequency (Fig. 6). Mechanistically, we proposed a conformational competition model in which a subpopulation of DNA undergoes a transcription-mediated conformational change that makes it unfavorable for R-loop formation (Fig. 7).

R-loop formation depends on RNAP and NTP concentration, which alters the rate of RNAP binding or the rate of RNA synthesis, respectively. The probability hypothesis provides an explanation for the results because the more chance that one DNA transcribes RNA per unit time, the more R-loop is expected to form based on Poisson distribution. Based on published sequencing data, this can also explain the correlation between R-loop distribution and transcription level (9, 27). However, it is noteworthy to emphasize that not all DNA can form an R-loop structure, that is, DNA's intrinsic property also regulates R-loop formation. For example, our previous study showed that a cMyc PQS at the non–template strand increases the R-loop formation because an accompanying G4 structure on the non–template strand stabilizes it. By contrast, a randomly scrambled cMyc PQS also formed an R-loop but significantly less than the cMyc PQS because of the lack of G4, suggesting that the R-loop formation is a tunable property that can be controlled by the DNA sequence and structures like G4 (37). While the sequence may modulate the energy required for RNA:DNA hybridization, the G4 structure can significantly suppress the rate of reannealing between the two complementary DNA strands. In addition, other factors, such as the location of the R-loop–prone motif to the promoter, DNA supercoiling, temperature, and the DNA substrate's length, can also alter the probability.

In our simulation model, we set R-loop formation as independent of individual events and determined by the probability. However, we evaluated other hypotheses in our prototype models (data not shown). For example, we assumed the DNA population initially had an energy or structural state distribution. This allows transitions between states after one transcription event for a single DNA molecule to determine whether it favored or disfavored R-loop formation. We also tested a model with multiple intermediate states, where the DNA that passed through more intermediates was more likely to form an R-loop. Although these complicated models could properly simulate the experimental data, the key point from all of them is determining the probability of R-loop formation after one transcription event for a single DNA molecule, rendering the "fake" states unnecessary. Moreover, we noted that all our tested simulation models approached complete R-loop formation given enough reaction time, which does not explain the plateau observed in our experimental data. This led us to propose the R-loop–inactive state to moderate the model. Since ensemble and single-molecule results show the same pattern, it is unlikely because of systematic errors. On the other hand, we can consider that the DNA alters the probability of R-loop formation during the transcription process. Hence, it is necessary to investigate further how and why some DNA remains in or gradually transitions to an R-loop–inactive state and whether this phenomenon generally affects other transcription experiments.

In summary, our investigation into the kinetics of R-loop formation during transcription reveals that both the frequency of RNAP loading and the transcription rate significantly influence R-loop formation. We demonstrate that R-loop formation is governed by the intrinsic probability of occurrence, modulated by transcriptional activity and density. These findings provide valuable insights into the regulatory mechanisms of R-loop formation and their implications for genomic stability. Understanding these dynamics is crucial for further understanding R-loops' roles in cellular processes and their potential impact on genome integrity.

## Experimental procedures

Detailed methods are provided in the Supporting Information.

### DNA preparation

DNA was labeled with Cy3 or Cy5 as previously described (37); details are provided in the supporting information. DNA was annealed in 10 mM Tris buffer (pH 8.3) with a biotinylated 18-mer primer at a ratio of 1:1.2:1.5 (NT:T: 18-mer). The DNA was heated to 95 °C for 5 min and then slowly cooled (1 °C/min) to room temperature.

### EMSA

Transcribed samples were run on a 10% polyacrylamide gel. The gel ran at 4 °C for 65 min, keeping the current constant at 12 mA per gel. The gel was stained for 10 min in 0.5X SYBER Green II RNA gel stain and destained in deionized water for 5 min. The gel was imaged at 628 nm fluorescence for 3 min and at Cy2 fluorescence for 3 s.

### smFRET assay

smFRET assays were performed using a home-built prism-type total internal reflection fluorescence microscope at room temperature (37, 40). Traces were recorded with 100 ms time resolution. PEG slides were pretreated with neutravidin (0.05 mg/ml), and labeled DNA was immobilized *via* biotin–neutravidin interaction. The imaging buffer used was prepared fresh, an oxygen scavenger system (1 mg/ml glucose oxidase, 0.8% v/v glucose, ∼10 mM Trolox, and 0.03 mg/ml catalase) was mixed with transcription buffer (commercial RNAP buffer, 40 mM Tris–HCl [pH 8.3], 50 mM KCl, murine RNase inhibitor 0.4 unit/μl). Transcription mix (imaging buffer containing RNAP and rNTPs) was flowed over the immobilized DNA.

### Ensemble smFRET measurements

Transcription experiments using varying RNAP and NTP titrations were performed. Measurements were collected every 10 min. Each FRET histogram was generated by collecting FRET values from at least 4000 molecules taken over 10∼20 movies. Molecules containing only donor (Cy3) signal were removed, and the histograms were analyzed with a Gaussian distribution function. Details on smFRET quantification are provided in the supporting information.

### Individual smFRET measurements

The 532 nm laser was used to excite DNA molecules. A transcription mix was injected into the chamber using a homemade flow system at 10 s to capture initial transcription events. To ensure the R-loop, low FRET signal was not because of Cy5 photobleaching, the 641 nm laser was turned on at the end of each movie. Transcription events were identified using a previously established PIFE method (37). Each PIFE peak, indicating a transcription event, was recorded. The time between the flow of the transcription mix and the initial transcription event was calculated along with the time between transcription events using a custom MATLAB script. Details on smFRET quantification are provided in the supporting information.

### Statistics for smFRET

In Figure 3, *B* and *E*, FE, and G dwell time between transcription events was counted from individual traces from independent molecules. The time between transcription events and the turnover rate (value ± SD) was calculated by fitting to a first-order exponential decay function. A Mann–Whitney test was performed as the curve is not a normal distribution.

### Simulation

The simulation was performed by home-made python code. The flow of stochastic simulation is depicted in Figure 6*A*. Two parameters, *k*_transcription_ and probability *p*, were given or varied to initiate the simulation. Thousand independent molecules were combined as one complete simulation. The *k*_transcription_ is the fitted initiation rate from single-molecule experiment (Fig. 4*B*). The transcription event was generated by exponential density function with *k*_transcription_ and randomly selected from 0.1 s to final time window subtracted 0.1 s, because the resolution of single-molecule experiment is 0.1 s. For example, given the time window *t*, the transcription event can happen at *t*_*1*_, between 0 and *t*-0.1 s, followed by a random function with probability *p* to determine the successful event of R-loop formation. If the event fails, the loop will regenerate a new event at *t*_*2*_, between *t*_*1*_ and *t*-0.1 s until R-loop forms or the *t*_*n*_ is outside the time window *t*. In Figure 6*B*, the *k*_transcription_ was fixed at 0.086 s, and the *p* was fitted to simulate the single-molecule data obtained in Figure 3*D*. The R-loop fraction was calculated by 10 independent simulations to determine the average ± standard error (95% confidence). In Figure 6*C*, both *k*_transcription_ and probability *p* were varied from 10^−5^ to 10^−1^ to initiate the simulations, and the R-loop fractions were computed by three independent simulations at each parameter pair. In Figure 5, *D*–*F*, all the data were generated from the results of Figure 6*B*, so each number was calculated by 10 independent simulations and showed as average ± standard error (95% confidence). In Figure 7*A*, *p* was fixed at 0.012, and the probability density function was generated for individual RNAP concentration to mimic the different binding rates with a time window of 10 min. In Figure 7*B*, *k*_transcription_ and *p* were fixed at 0.086 s^−1^ and 0.012, respectively, but the time window was varied. For Figure 7*C*, the simulation was generated and fitted as ensemble reaction, which was coded by partial differential equation and optimization fitting function with python packages. We noted that simulation-generated figures vary in the number but maintain the same pattern and significance.

## Data availability

All the data are contained and mentioned in the article and supporting information. The raw data and gels are provided in the Data Source file.

## Supporting information

This article contains supporting information.

## Conflict of interest

The authors declare that they have no conflicts of interest with the contents of this article.

## Supporting information

SUPPLEMENTAL_revision_jim_v2.docx

SUPPLEMENTARY TABLE.docx
