# Supplementary material for: High-frequency transcription leads to rapid R-loop formation
Source: J Biol Chem. 2025 Apr 16;301(6):108514. doi: 10.1016/j.jbc.2025.108514 (PMC12137161; doi:10.1016/j.jbc.2025.108514)
Supplement: SUPPLEMENTAL_revision_jim_v2.docx [file mmc1.docx]

S**upporting Information**

High frequency transcription leads to rapid R-loop formation

Bradleigh Palmer^§1^, Chun-Ying Lee^§2^, Leya Yang^2^, Tapas Paul^2^ and Sua Myong^1, 2^

1. Department of Biophysics, Johns Hopkins University, Baltimore, Maryland 21218, USA

2. Program in Cellular and Molecular Medicine, Boston Children’s Hospital, Boston, Massachusetts, USA

§ These authors contributed equally to this work

*Correspondence: [sua.myong@childrens.harvard.edu](mailto:smyong@jhu.edu)

Table of Contents

Materials and methods 1

Chemicals 1

Enzymes: 2

Oligonucleotides: 2

DNA Preparation: 3

smFRET assay: 3

smFRET Quantification: 3

EMSA: 4

Simulation of NTP hydrolysis: 4

Supplementary Fig. 1 6

Supplementary Fig. 2 7

References 8

Materials and methods

**Chemicals:**

Cy3-NHS-ester and Cy5-NHS-ester were purchased from GE Healthcare.

Ammonium persulfate was purchased from Millipore Sigma.

40% Acrylamide/Bis Solution, 29:1, 10x TBE Buffer, and TEMED were purchased from Bio-Rad.

Low molecular weight DNA ladder and gel loading dye (Purple 6X, no SDS) were purchased from New England Biolabs.

SYBR Green II RNA Gel Stain, 10,000X concentrate in DMSO was purchased from Thermofisher. Glucose oxidase (Sigma, 9001-37-0), Trolox (Sigma, 53188-07-1), Glucose, Potassium chloride, EDTA, Tris, Sodium hydroxide (Thermofisher).

**Enzymes:**

All enzymes were purchased from New England Biolabs.

**Oligonucleotides:**

DNA oligos were designed in our previous work (1) and purchased from Integrated DNA Technologies (IDT). The sequences are written from 5’ to 3’. Cy3-T and Cy5-T mean the dye was labeled on a modified Thymine base, and iCy3 means the dye was labeled on the backbone.

Biotin- 18mer:

GCCTCGCTGCCGTCGCCA-Biotin

FRET construct for Figure 1, 2 and 3:

Top:

TGGCGACGGCAGCGAGGCTAAATTAATACGACTCACTATAGGGAGACCACAACG/Cy3-T/TAGGGTGGGTAGGGTGGGTTATCAGCTCCAGGTCT

Bottom:

AGACCTGGAGCTGA/Cy5-T/AACCCACCCTACCCACCCTAACGTTGTGGTCTCCCTATAGTGAGTCGTATTAATTTA

FRET construct for Figure 4:

Top:

TGGCGACGGCAGCGAGGCTAAATTAATACGACTCAC/Cy3-T/ATAGGGAGACCACAACGTTAGGGTGGGTAGGGTGGG TTATCAGCTCCAGGTCT

Bottom:

AGACCTGGAGCTGATAACCCACCCTACCCACCC/Cy5-T/AACGTTGTGGTCTCCCTATAGTGAGTCGTATTAATTTA

FRET construct for Figure 5:

Top:

TGGCGACGGCAGCGAGGCTAAATTAATACGACTCACTATAGGGAGACCACAACG/Cy3-T/TAGGGTGGGTAGGGTGGG TTATCAGCTCCAGGTCT

Bottom:

AGACCTGGAGCTGATAACCCACCCTACCCACCC/Cy5-T/AACGTTGTGGTCTCCCTATAGTGAGTCGTATTAATTTA

FRET construct for Figure S1:

Top:

TGGCGACGGCAGCGAGGCTAAATTAATACGACTCACTATAGGGAGACCACAACG/iCy3/TAGGGTGGGTAGGGTGGGT/Cy5-T/ATCAGCTCCAGGTCT

Bottom:

AGACCTGGAGCTGATAACCCACCCTACCCACCCAACGTTGTGGTCTCCCTATAGTGAGTCGTATTAATTTA

**DNA Preparation:**

DNA oligonucleotides have an internal amine modification, which was used for Cy3 or Cy5 labeling. Oligonucleotides were dissolved in H_2_O to create a 100 μM stock. 25 μL of DNA was mixed with 0.1 mg Cy3-NHS-ester or Cy5-NHS-ester (GE Healthcare), 5 μL 1 M sodium bicarbonate buffer (freshly prepared), and 25 μL H_2_O, for a final volume of 50 μL and final DNA concentration of 50 μM. The mixture was kept in the dark and rotated at 40 °C for 6h. Two rounds of ethanol precipitation were done to remove excess dye. Ethanol precipitation was done by mixing the 50 μL reaction with 125 μL of cold 100% ethanol and 3 μL 5 M sodium chloride. The mixture was cooled at −80 °C for 1 h, then centrifuged at 4 °C at 15,000 r.p.m. for 30min. The DNA pellet was washed twice with cold 70% ethanol. After two rounds of ethanol precipitation, the labeled DNA was resuspended in 25 μL 10 mM Tris buffer.

**smFRET assay:**

smFRET assays were performed using a home-built prism-type total internal reflection fluorescence microscope at room temperature (1, 2). Solid-state 532nm and 641nm lasers were used to take single-molecule measurements. smCamera software was used to record single-molecule traces with 100ms time resolution. Traces were analyzed using Interactive Data Language. Custom MATLAB scripts were used to generate trajectories and FRET histograms. PEG slides were pretreated with neutravidin (0.05 mg/mL) and 10 nM labeled DNA was diluted to 25 pM and immobilized. The imaging buffer used was prepared fresh, an oxygen scavenger system (1 mg/mL glucose oxidase, 0.8% v/v glucose, ~10 mM Trolox, and 0.03 mg/mL catalase) was mixed with transcription buffer (commercial RNAP buffer, 40 mM Tris-HCl pH 8.3, 50 mM KCl, murine RNase inhibitor 0.4 unit/µL). Transcription mix (imaging buffer containing RNAP and rNTPs) was flowed over the immobilized DNA.

**smFRET Quantification:**

OriginPro 8.5 was used to create histograms and fit curves for single-molecule experiments. FRET histograms were generated by collecting FRET values from at least 4000 molecules taken over 10~20 movies. Molecules containing only donor (Cy3) signal were removed and the histograms were analyzed with a Gaussian distribution function. R-loop fraction calculated from various time points was plotted and an exponential function was used to fit the curves. R-loop fraction calculated from the histograms of various RNAP and NTP titrations was plotted, and a Hill function was used to fit the curves. Dwell time between transcription events was counted from individual traces from independent molecules. The time between transcription events was binned and plotted. RNAP turnover rate (value ± SEM) was calculated by fitting to a first-order exponential decay function.

**EMSA:**

Transcription experiments were conducted using dsDNA that is only labeled with Cy5. Each transcription condition was performed using a 20 µL reaction. The transcription mix is added to the DNA (10nM final concentration), and then NTPs are added to initiate the reaction. The transcription mix consists of T7 RNAP, RNAP buffer, 50 mM KCl, 0.02 unit/µL TIPP, and 0.4 unit/µL RNase inhibitor. rNTPs are added last at the appropriate concentration. The 0 min time points do not include NTPs. Reactions were run at 25 °C. Transcription was quenched by adding 0.5 µL of 0.5 M EDTA at each time point. The reaction for RNase-treated samples was stopped with 1 μM T7 promoter DNA, top and bottom strand annealed in 10mM Tris buffer (pH 8.3). Then 0.125 u/μL RNase H was added to the reaction for 15 min at 25 °C. After RNase H digestion, samples were quenched with EDTA. The samples were mixed with 4 µL 50% glycerol and 0.1% SDS before loading on the gel. The transcribed samples were loaded onto a 10% polyacrylamide gel containing 10% acrylamide/bis- acrylamide solution, 1X TBE buffer, 1% APS, and 1% TEMED. The gel was run at 4$℃$for 65 minutes, with a constant current of 12 mA per gel. The gel was stained for 10 minutes in 0.5X SYBER Green II RNA gel stain (2.5 µl of 10,000 X SYBR™ Green II RNA Gel Stain concentrate, 50 mL deionized water) then destained in deionized water for 5 minutes. The gel was imaged at 628 nm fluorescence for 3 minutes and at Cy2 fluorescence for 3 seconds.

**Simulation of NTP hydrolysis:**

The simulation was performed by home-made python code. The NTP hydrolysis rate was simulated using a Michaelis-Menton model adapted from previous studies (3). The hydrolysis rate (*k_cat_*) was determined as:

$$k_{cat, hydrolysis}=\frac{V_{max}\cdot[NTP]}{K_{m}+[NTP]}$$

Where K_m_ = 76 µM and *V_max_* = 220 s^-1^, values observed in the presence of DNA and obtained from literature(3, 4). The NTP concentration was dynamically updated every minute (0 to 60 min) based on the equation:

$${[NTP]}_{t}={[NTP]}_{0}-t\cdot\left[ DNA:RNAP \right]\cdot{Length}_{RNA}\cdot<n>$$

Where [NTP]_0_ (from 10 to 1000 µM) and [DNA:RNAP] (100 pM and 10 nM) were initial conditions, RNA length (L) was set to 50 nt as our designed construct, and the average event number (<n>) was 9.5, based on single-molecule experiment (Fig. 4C). This approach allowed both NTP concentration and hydrolysis rate to change over time.


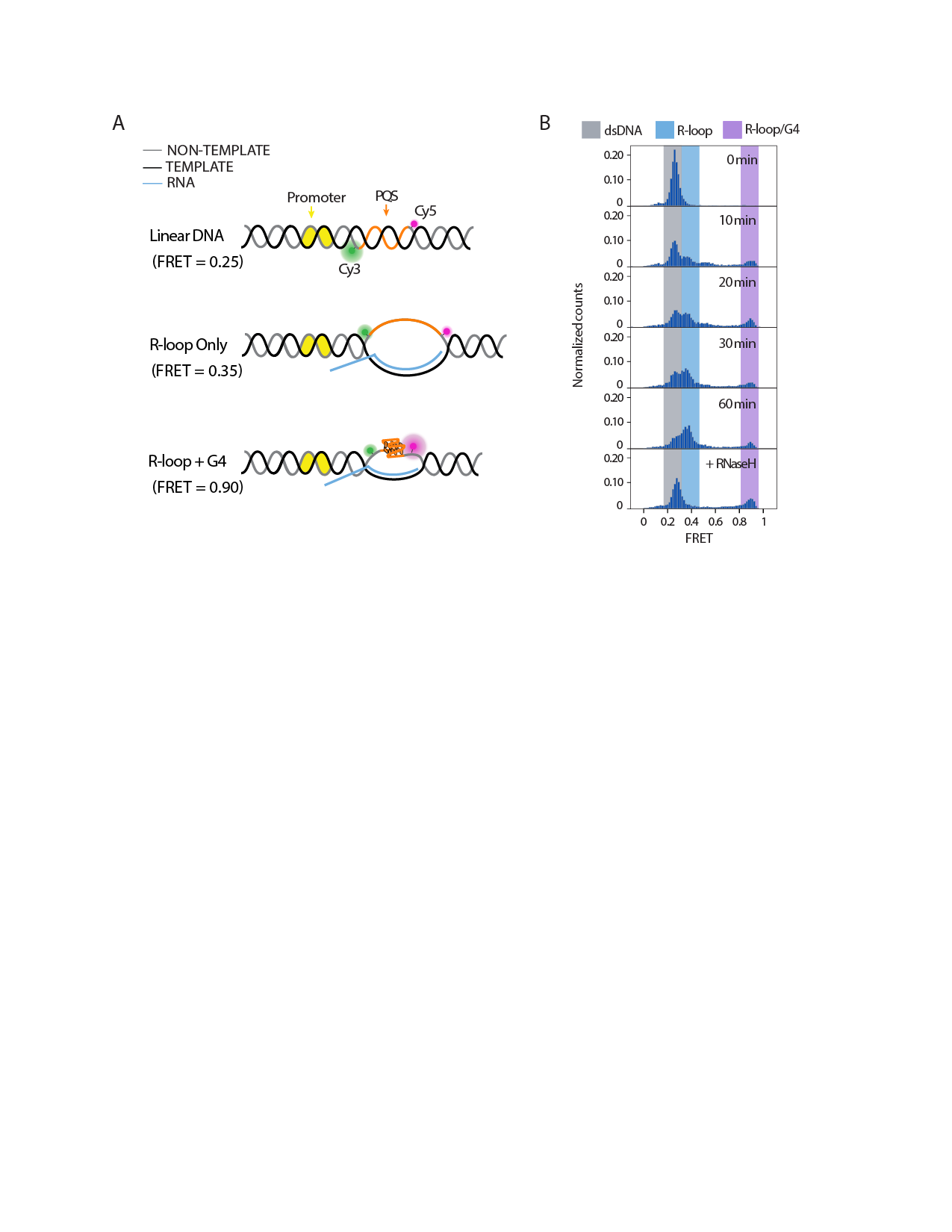


Supplementary Fig. 1 *FRET construct for visualizing R-loop and G4 formation.* (a) FRET construct produces a 0.25 FRET value when DNA is linear, upon R-loop formation the dyes are closer together producing a 0.35 FRET, and the dyes are at their closest upon G4 folding producing a FRET value of 0.9. (b) FRET histogram with transcription incubation intervals at 10, 20, 30, and 60 min and 10 min RNase H incubation. In (b) a single representative histogram is shown from n=3 independent measurements. 1 mM NTP and 1 µM RNAP were used for transcription.


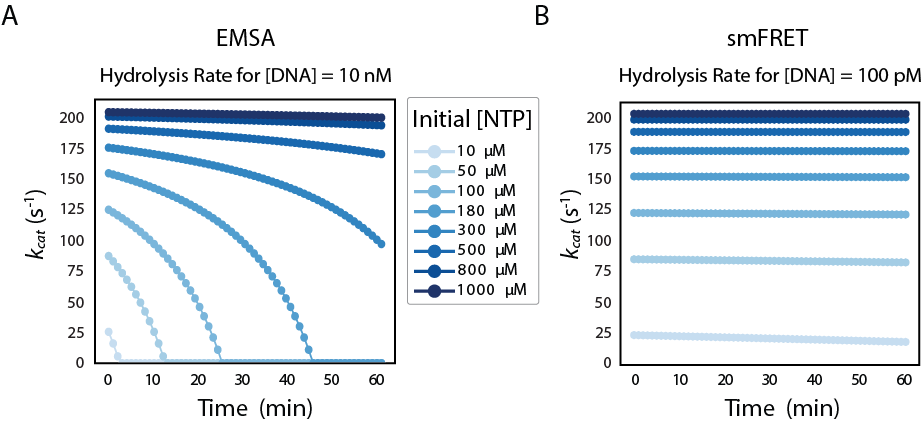


# Supplementary Fig. 2 *Simulation of NTP hydrolysis rate.* (a) global NTP hydrolysis rate by 10 nM DNA. (b) global NTP hydrolysis rate by 100 pM DNA. Each condition represents the EMSA and smFRET experiment, respectively

References

1. Lee, C. Y., McNerney, C., Ma, K., Zhao, W., Wang, A., andMyong, S. (2020) R-loop induced G-quadruplex in non-template promotes transcription by successive R-loop formation Nat Commun **11**, 3392 10.1038/s41467-020-17176-7

2. Roy, R., Hohng, S., andHa, T. (2008) A practical guide to single-molecule FRET Nature Methods **5**, 507-516 10.1038/nmeth.1208

3. Anand, V. S., andPatel, S. S. (2006) Transient state kinetics of transcription elongation by T7 RNA polymerase J Biol Chem **281**, 35677-35685 10.1074/jbc.M608180200

4. Arnold, S., Siemann, M., Scharnweber, K., Werner, M., Baumann, S., andReuss, M. (2001) Kinetic modeling and simulation of in vitro transcription by phage T7 RNA polymerase Biotechnol Bioeng **72**, 548-561, <https://www.ncbi.nlm.nih.gov/pubmed/11460245>
