# Supplementary material for: High-frequency transcription leads to rapid R-loop formation
Source: J Biol Chem. 2025 Apr 16;301(6):108514. doi: 10.1016/j.jbc.2025.108514 (PMC12137161; doi:10.1016/j.jbc.2025.108514)
Supplement: SUPPLEMENTARY TABLE.docx [file mmc2.docx]

S**upplementary table**

High frequency transcription leads to rapid R-loop formation

Bradleigh Palmer^§1^, Chun-Ying Lee^§2^, Leya Yang^2^, Tapas Paul^2^ and Sua Myong^1, 2^

1. Department of Biophysics, Johns Hopkins University, Baltimore, Maryland 21218, USA

2. Program in Cellular and Molecular Medicine, Boston Children’s Hospital, Boston, Massachusetts, USA

§ These authors contributed equally to this work

*Correspondence: [sua.myong@childrens.harvard.edu](mailto:smyong@jhu.edu)

Table S1: Molecule counts for single-molecule analysis

| **Condition** | **R-loop Formation** | **# of Molecules** | **N (Independent Measurements)** | **Experiment** |
| --- | --- | --- | --- | --- |
| 500 µM NTP 1 µM RNAP | No | 317 | 3 | NTP Titration |
| 500 µM NTP 1 µM RNAP | Yes | 34 | 3 | NTP Titration |
| 825 µM NTP 1 µM RNAP | No | 222 | 3 | NTP Titration |
| 825 µM NTP 1 µM RNAP | Yes | 37 | 3 | NTP Titration |
| 1mM NTP 750 nM RNAP | No | 290 | 3 | RNAP Titration |
| 1mM NTP 750 nM RNAP | Yes | 24 | 3 | RNAP Titration |
| 1mM NTP 825 nM RNAP | No | 205 | 3 | RNAP Titration |
| 1mM NTP 825 nM RNAP | Yes | 21 | 3 | RNAP Titration |
| 1mM NTP 900 nM RNAP | No | 265 | 3 | RNAP Titration |
| 1mM NTP 900 nM RNAP | Yes | 25 | 3 | RNAP Titration |
| 1mM NTP 1 µM RNAP | No | 1171 | 19 | NTP Titration and RNAP Titration |
| 1mM NTP 1 µM RNAP | Yes | 255 | 19 | NTP Titration and RNAP Titration |
| 1mM NTP 1 µM RNAP | N/A | 344 | 2 | Initiation |
